# Examining predictors of loneliness among Older Americans Act National Family Caregiver Support Program participants

**DOI:** 10.3389/fpubh.2024.1337838

**Published:** 2024-07-01

**Authors:** Heather L. Menne, Claire Pendergrast

**Affiliations:** ^1^Department of Sociology and Gerontology, Miami University, Oxford, OH, United States; ^2^Sociology Department, Syracuse University, Syracuse, NY, United States

**Keywords:** caregiving, caregiver support, loneliness, hierarchical multivariate regression, Older Americans Act

## Abstract

Family caregivers may be at particular risk for social isolation and loneliness. Multiple factors can impact caregivers’ health and well-being outcomes, including loneliness. Guided by an adaptation of the Stress Process Model of Caregiving, this study uses the 2019 National Survey of Older Americans Act Participants (NSOAAP)-Family Caregiver Support module to inform efforts to reduce loneliness through family caregiver support programs. A hierarchical multiple regression model reveals that caregivers who report more loneliness are more likely to be female, Hispanic, living alone, not a child or other caregiver of the care recipient, have a care recipient with 3+ ADL needs, experience more social life conflict related to caregiving, experience less joy in caregiving, feel less appreciated by the care recipient, feel less support in caregiving, and attend counseling. This study helps advance the goals of the National Strategy to Support Family Caregivers, and the findings underscore the importance of continuing and expanding efforts to address loneliness and related well-being outcomes among family caregivers.

## Introduction

Social isolation and loneliness are known public health threats. Over 30 percent of adults over the age of 45 feel lonely, and almost 25 percent of adults over the age of 65 are considered socially isolated (1). A population who may be at particular risk for social isolation and loneliness are family caregivers, who may be spouses, partners, or adult children providing a range of unpaid care for older family members. The literature on family caregivers points to multiple factors that can impact a caregiver’s health and well-being outcomes, including loneliness. For example, research by Robison et al. (2) found that caregivers who live with their care recipient are 2.5 times as likely to report feeling isolated, compared with caregivers who do not live with their care recipient, and caregivers with ongoing unmet long-term service and support (LTSS) needs are 3.8 times as likely to report feeling isolated. Research also indicates that caregivers of people living with dementia (PLWD) report greater rates of loneliness (3), and this may be due in part to the loss of the PLWD’s memory function as a resulting barrier to social interactions (4).

The 2000 reauthorization of the Older Americans Act included the establishment of the National Family Caregiver Support Program (NFCSP), the first comprehensive federal program with the purpose of supporting the needs of family caregivers (5). Delivered through Area Agencies on Aging, the core services of the NFCSP provides information to caregivers about available services; assistance in gaining access to services; counseling, support groups, and caregiver training; respite care; and supplemental services. These services are made available to caregivers caring for individuals 60 years of age and older or individuals of any age with Alzheimer’s disease and related disorders, and older relative caregivers to children under the age of 18 or adults ages 18–59 with disabilities (5). Nationwide, over 800,000 caregivers received services through the NFCSP in 2019.

In collaboration with other federal agencies, the Administration for Community Living released the first National Strategy to Support Family Caregivers in 2022 (6). The National Strategy seeks to support those providing care across the life course. In relation to loneliness of NFCSP caregivers, the National Strategy includes goals to strengthen services and supports (Goal 3) and expand data, research, and evidence-based practices (Goal 5). The result of this study informs both goals since to date there has been no empirical investigation into the role of the NFCSP on caregiver loneliness. This study provides a unique opportunity to expand our understanding of the role of the NFCSP on caregiver experiences. In addition, the 2019 data collection of the National Survey of Older Americans Act Participants was the first to measure loneliness with the 3-item version of the UCLA loneliness scale (7).

### Conceptual model

This study examines the impact of caregiver and care recipient characteristics on the loneliness of the caregivers receiving OAA NFCSP services. This work is guided by an adaptation of the Stress Process Model of Caregiving [SPMC; (8)] which includes background characteristics, primary stressors, secondary stressors, and mediators/caregiver supports as predictors of outcomes (e.g., loneliness). Background and context variables are those sociodemographic characteristics (e.g., age, race, gender, and education level) or related context variables (e.g., overall health) of an individual that may contribute either directly or indirectly to the experience of primary stressors, secondary stressors, or the outcomes of caregiving. Using data from the National Study of Caregiving, Parr and Mielenz (9) demonstrate that caregiving outcomes related to caregiving gains and purpose in life are moderated by race. Analysis by Bramboeck et al. (10) in a study of dementia caregivers shows that male gender of caregivers and living with the person who has dementia are significant predictors of loneliness.

For caregiving, primary stressors can include variables about the care recipient’s such as number of activities of daily living (ADL) needs or having a dementia diagnosis. Research demonstrates an association between care recipients’ functional abilities and the well-being of caregivers (11, 12). Pearlin et al. (8), when describing secondary stressors, note that “an underlying premise of our conceptual scheme is that one set of stressors can lead to another” (p. 588). Secondary stressors can be the roles or psychological attributes that are enhanced or compromised due to caregiving (e.g., caregivers’ experiences of joy related to caregiving social engagement). Pearlin et al. (13) demonstrated the value of secondary stressors, and specifically work strain and the constriction of leisure activities, on depression among caregivers to people with AIDS.

While caregivers will experience the stressors of caregiving in myriad ways, the mediators are those factors which are often assessed to understand caregiving outcomes among the range of experiences (8). A principal mediator is social support, which can be measured by the existence or type of services used by caregivers. For example, a study of loneliness among caregivers of people living with Parkinson’s disease reveals that caregivers attending support groups reported less loneliness (14).

**Figure 1:**
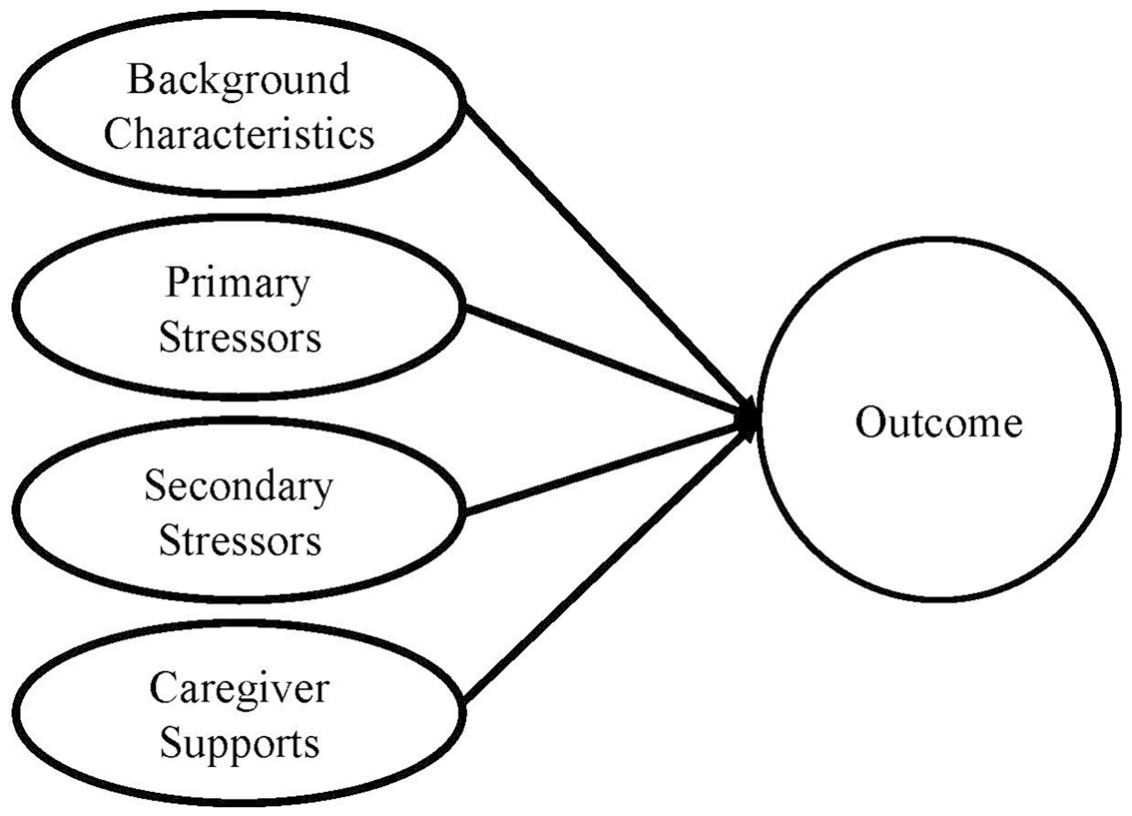
Adapted stress process model of caregiving.

Leveraging the SPMC, this study seeks to identify factors that predict loneliness among family caregiver support program recipients. Figure 1 is the adapted SPMC guiding these analyses.

## Methods

### Data source

The Administration on Aging within the Administration for Community Living conducts the National Survey of Older Americans Act Participants (NSOAAP) to measure service and program quality and learn more about OAA program participants (15). For this study, we used the 2019 NSOAAP-Family Caregiver Support module, which contains responses from nearly 2,000 NFCSP caregivers. The process to reach the sample of 2,000 NFCSP caregivers first required selecting a sample of area agencies on aging (which are NFCSP providers), and from those agencies selecting a sample of NFCSP caregivers. These steps are taken to achieve a nationally representative sample of NFCSP participants (15). Through a computer-assisted telephone interview (CATI), respondents answered questions related to demographic and socioeconomic characteristics, caregiving experience, well-being, program satisfaction, caregiving intensity, unmet needs, and service usage. The NSOAAP-Family Caregiver Support module includes filter questions which remove NFCSP caregivers from completing the module if they are not caring for an older adult (7).

### Measures

Using the SPMC as a guide, we chose measures available from the NSOAAP (see Table 1).

**Table 1:** Characteristics of study population.

	% (n)	Average loneliness score
Gender		
Male	26.6% (399)	5.06^
Female	73.3% (1090)	5.38
Race/ethnicity		
Non-Hispanic white	69.7% (1172)	5.31
Non-Hispanic black	18.5% (180)	5.05
Hispanic	8.3% (85)	5.82
Non-Hispanic other	3.6% (52)	5.17
Age		
18–64	40.5% (588)	5.10^
65+	59.5% (901)	5.43
Educational attainment		
High school degree or less	68.9% (1004)	5.37
Some college degree or more	31.1% (485)	5.14
Living arrangement		
Lives alone	75.1% (1126)	5.19^
Lives with others	24.9% (363)	5.61
Relationship to care recipient		
Spouse	44.1% (662)	5.54
Child	42.1% (642)	5.21
Other	13.7% (185)	4.78
Rurality		
Urban	36.2% (551)	5.23
Suburban	27.3% (366)	5.27
Rural	36.5% (572)	5.38
Caregiver self-rated health		
Fair/Poor	83.1% (1263)	5.41**
Good/Very good/Excellent	16.9% (226)	4.76
Care recipient dementia		
Dementia	60.1% (856)	4.95**
No dementia	39.9% (633)	5.52
ADL impairments		
0–2 ADL impairments	31.3% (515)	4.54***
3+ ADL impairments	68.7% (974)	5.65
Social conflicts with caregiving	34.0% (514)	6.56***
No social conflicts with caregiving	66.0% (975)	4.64
Joy due to caregiving	56.6% (867)	5.00***
No joy from caregiving	43.4% (622)	5.69
Appreciation for caregiving	70.1% (1045)	4.99***
No appreciation for caregiving	29.9% (444)	6.02
Has enough support	60.9% (935)	4.67***
Does not have enough support	39.1% (554)	6.25
Training services	28.7% (420)	5.42
No training services	71.3% (1069)	5.24
Counseling services	23.2% (329)	5.71
No counseling services	76.8% (1160)	5.17**
Support group services	28.7% (413)	5.37
No support group services	71.3% (1076)	5.27
Loneliness score (SD)		5.3 (2.05)

^*p* < 0.10, *p < 0.05, **p < 0.01, ***p < 0.001. *n* = number of participants = 1,489; SD = standard deviation; all numbers are unweighted and percentages and means are weighted. Adjusted Wald tests were used to assess differences in loneliness between groups.

#### Outcome

Loneliness was measured using the 3-item version of UCLA loneliness scale. Respondents were asked how often they feel that they lack companionship, feel left out, and feel isolation from others (response options 1 = “hardly ever” 2 = “some of the time” and 3 = “often”). Caregiver responses to these three items were summed to create an index of 3–9 with higher numbers indicating more loneliness.

#### Background and context

Sociodemographic background and context variables are based on caregiver self-reported survey responses. Variables included age (0 = 64 and younger; 1 = 65 and older), gender (0 = male; 1 = female), educational level (0 = High School diploma or less; 1 = some college or above), income (0 = less than $20,000; 1 = $20,000 or more), geographic location/rurality (0 = urban; 1 = suburban; 2 = rural), living arrangement (0 = lives with others; 1 = lives alone), and relationship to care recipient (0 = spouse; 1 = child; 2 = other). Because race and ethnicity were asked as unique questions for each category, these were combined to yield a race/ethnicity variable (0 = Non-Hispanic White, 1 = Non-Hispanic Black; 2 = Hispanic; 3 = Non-Hispanic Other Race).

Caregiver health is included as a context variable anticipated to have a direct effect on loneliness but occurring separate from the caregiving experience. Caregiver health is based on a 1-item indicator dichotomized for poor/fair health (0) and good/very good health (1).

#### Primary stressors

The primary stressors are assessed by objective measures related to the care recipient and reported by the caregiver. Specifically included was whether a care recipient had received a doctor’s diagnosis of a memory related disease such as dementia (0 = no dementia diagnosis; 1 = dementia diagnosis). A dichotomous variable on care recipient’s activities of daily living (ADL) needs was also computed based on whether the care recipient had need of help with task such as dressing, eating, and bathing. Caregivers provided yes or no responses to six questions about care needs, these six were then summed and dichotomized to represent care recipients with 0–2 needs (0) and 3+ needs (1).

#### Secondary stressors

Variables operationalized to represent secondary stressors include caregiver-reported subjective measures of experiencing joy in caregiving (0 = sometimes, rarely, or never; 1 = usually or always), feeling appreciated by the care recipient (0 = sometimes, rarely, or never; 1 = usually or always), feeling enough support (0 = sometimes, rarely, or never; 1 = usually or always), and experiencing social life conflicts related to caregiving (0 = sometimes, rarely, or never; 1 = usually or always).

#### Mediators/caregiver supports

Mediators represent those caregiver services which may lessen experiences of loneliness. Specifically, measures were caregiver reports of attending caregiver education or training classes (0 = no; 1 = yes), receiving counseling (0 = no; 1 = yes), and attending support groups (0 = no; 1 = yes).

### Analysis plan

To facilitate our understanding of what caregiver and care recipient characteristics contribute to the loneliness of caregivers using community support services, our analysis plan proceeds in two stages. First, we assessed differences between groups based on average scores on the loneliness outcome using adjusted Wald tests. Next, we used hierarchical multivariate regression [or blockwise selection; (16)] to determine the significance of the independent variables in predicting caregiver loneliness. Hierarchical multiple regression provides a test of statistical significance after the addition of each predetermined block of variables (e.g., background and context and primary stressors), denoting which blocks significantly contribute to the final prediction of the outcome. The increased contribution of each block of variables is represented in the R^2^-change value and its related significance.

Among the 1,909 respondents who completed the survey, the listwise deletion method was used for missing observations and the sample weighted and analyzed in this article included 1,489 respondents. To ensure our results were not skewed by using listwise deletion, additional analyses revealed that there were not statistically significant differences in key demographic characteristics between the 420 respondents removed after listwise deletion and the respondents in the final sample. Weights were applied to reflect the probability sampling methodology used in the survey, and are used to create a dataset that is nationally representative of NFCSP participants who are caregivers for older adults. All analyses used weighted survey data and were conducted using Stata version 16.1.

## Results

### Descriptive characteristics

Details on the NFCSP respondents can be found in Table 1. The majority of the sample was female (73.3%), age 65 and older (59.5%), non-Hispanic White (69.7%), with a high school degree or less (68.9%), and living alone (75.1%). There was a balanced distribution of caregivers living in urban (36.2%), suburban (27.3%), and rural communities (36.5%). Caregivers were often the spouse to the care recipient (44.1%) or the child of the care recipient (42.1%). Six out of 10 caregivers were caring for someone living with dementia (60.1%); and more than two-thirds of caregivers were caring for someone with 3+ ADL needs (68.7%). Despite participating in the NFCSP, the majority of caregivers reported not attending caregiver education or training classes (71.3%), receiving counseling (76.8%), or attending support groups (71.3%).

### Significant differences between groups on the loneliness outcome

Participants in the NFCSP reported an average loneliness score of 5.3 (*SE* = 0.088). There were significant differences in loneliness scores for selected caregiver characteristics (see Table 1). For example, caregivers who reported fair/poor self-rated health had higher levels of loneliness (5.41) than those reporting good/very good/excellent health (4.76; *p* < 0.01). Caregivers to people living with dementia have less loneliness (4.95) compared to those caring for someone without dementia (5.52; *p* < 0.01). Caregivers to people with 3+ ADL needs were reported more loneliness (5.65) than caregivers to people with 0–2 ADL needs (4.54; *p* < 0.001).

In addition, there were significant differences in loneliness based on secondary stressors and mediators/caregivers supports. Caregivers who reported social conflicts with caregiving (6.56; *p* < 0.001), no joy from caregiving (5.69; *p* < 0.001), no feelings of appreciation from the care recipient (6.02; *p* < 0.001), and not feeling enough support (6.25; *p* < 0.001) also reported higher loneliness scores. In reviewing the mediators/caregiver supports, caregivers receiving counseling (compared to those not receiving counseling) reported higher loneliness scores (5.71; *p* < 0.01).

### Multiple regression predicting the loneliness of caregivers

We used hierarchical multivariate regression to determine the significance of the independent variables and SPMC model components in predicting loneliness of caregivers (see Table 2). The results of the four models predicting loneliness scores indicate that the *R*^2^ value increases significantly with Steps 1, 2, 3, and 4 (*R*^2^ = 0.36, *p* = 0.00).

**Table 2:** Regression results predicting caregiver loneliness.

	Model 1. β (*SE B*)	Model 2. β (*SE B*)	Model 3. β (*SE B*)	Model 4. β (*SE B*)
Gender (ref: male)	0.54 (0.18)**	0.51 (0.17)**	0.34 (0.15)*	0.32 (0.15)*
Non-Hispanic Black (ref: NHW)	−0.13 (0.26)	−0.27 (0.25)	−0.14 (0.22)	−0.16 (0.23)
Hispanic (ref: NHW)	0.68 (0.26)^	0.49 (0.25)*	0.55 (0.26)*	0.52 (0.24)*
Non-Hispanic Other (ref: NHW)	0.07 (0.34)	0.08 (0.37)	0.13 (0.46)	0.05 (0.44)
Age (ref: <65)	0.22 (0.23)	0.13 (0.22)	0.18 (0.18)	0.18 (0.18)
Educational attainment (re: HS or less)	−0.15 (0.18)	−0.10 (0.18)	−0.10 (0.15)	−0.13 (0.15)
Living arrangement (ref: lives with others)	0.39 (0.22)^	0.39 (0.21)^	0.37 (0.18)*	0.34 (0.18)^
Relationship to care recipient: Child (ref: spouse)	−0.27 (0.24)	−0.25 (0.24)	−0.35 (0.19)^	−0.34 (0.19)^
Relationship to care recipient: Other (ref: spouse)	−0.82 (0.26)**	−0.76 (0.26)**	−0.75 (0.20)***	−0.73 (0.20)***
Suburban (ref: urban)	0.03 (0.22)	−0.03 (0.21)	0.05 (0.15)	0.06 (0.17)
Rural (ref: urban)	0.18 (0.20)	0.09 (0.19)	0.11 (0.18)	0.11 (0.18)
Caregiver self-rated health (ref: fair/poor)	−0.67 (0.20)**	−0.58 (0.19)**	−0.18 (0.20)	−0.22 (0.18)
Care recipient dementia (ref: no dementia)		0.50 (0.16)**	0.17 (0.14)	0.11 (0.14)
ADL impairments (ref: 0–2 ADLs)		0.98 (0.16)***	0.49 (0.14)**	0.52 (0.14)***
Social conflicts due to caregiving (ref: never, rarely or sometimes has caregiving conflict with social life)			1.38 (0.17)^a^***	1.35 (0.17)^a^***
Joy due to caregiving (ref: never, rarely or sometimes feels joy due to caregiving)			−0.55 (0.15)***	−0.55 (0.15)***
Appreciation for caregiving (ref: never, rarely or sometimes feels appreciated by care recipient)			−0.45 (0.16)**	−0.44 (0.16)**
Has enough support (ref: does not feel like they have enough support)			−0.93 (0.16)***	−0.95 (0.16)***
Training services (ref: no training services)				0.18 (0.18)
Counseling services (ref: no counseling services)				0.43 (0.18)*
Support group services (ref: no support group services)				−0.10 (0.18)
Constant	4.95 (0.31)***	4.10 (0.33)	5.38 (0.32)***	5.31 (0.32)***
Adjusted *R*^2^	0.062	0.128	0.347	0.356
Δ *R*^2^		0.646	0.221	0.009
Δ *F*		24.97***	25.28***	−52.48^

^a^Due to the standardized estimate being greater than one several steps were taken to assess sources of multicollinearity. Pairwise correlations were examined between all variables in the model, and no correlations greater than 0.49 (Social Conflicts due to Caregiving and Loneliness) were found to suggest the possibility of multicollinearity. In addition, in reviewing the VIF and TOL values, no concerns with multicollinearity were observed (highest VIF = 1.81, lowest TOL = 0.55). No attempt was made to respecify the model simply to eliminate the higher-than-usual coefficient. As Deegan (17) suggests, given standardized coefficients greater than one can legitimately occur, there is no compelling reason to re-specify a given model. ^*p* < 0.10, *p < 0.05, **p < 0.01, ***p < 0.001. NHW, non-Hispanic White; HS, high school; ADLs, activities of daily living.

The overall interpretation of Model 4 indicates that 11 predictors significantly contribute to the *R*^2^ value of 0.36 (*p* = 0.00). This model suggests that caregivers who report more loneliness are female (*β* = 0.32, *p* = 0.03), Hispanic (*β* = 0.52, *p* = 0.03), living alone (*β* = 0.34, *p* = 0.06), not a child (*β* = −0.34, *p* = 0.08) or other caregiver (*β* = −0.73, *p* < 0.001), have a care recipient with 3+ ADL needs (*β* = 0.52, *p*<0.001), experience more social life conflict related to caregiving (*β* = 1.35, *p* < 0.001), experience less joy in caregiving (*β* = −0.55, *p* < 0.001), feel less appreciated by the care recipient (*β* = −0.44, *p* = 0.01) feel less support in caregiving (*β* = −0.95, *p* < 0.001), and attend counseling (*β* = 0.43, *p* = 0.02).

## Discussion

The population represented here are a unique set of caregivers who participated in the Older Americans Act NFCSP services. The research literature has long identified that caregivers experience myriad forms of stress, strains, and poor outcomes (2, 3, 10–14) and that many caregivers benefit from the use of supportive services, such as offered through the NFCSP (18). The intent of this study was to understand the experience of loneliness among family caregiver support program participants, and identify factors which may contribute to lower levels of loneliness.

Guided by an adapted version of the SPMC (8), the analyses explored the background characteristics, primary stressors, secondary stressors, and mediator/caregiver support variables predicting loneliness. Among the background characteristics of caregivers, group differences were seen based on caregiver self-reported health, with caregivers who reported fair/poor self-rated health having higher levels of loneliness than those reporting good/very good/excellent health. Caregivers self-rated health was significant in early models of the hierarchical multivariate regression, but it was not significant in the final Model 4. This suggests that poor caregiver health leads to increased loneliness because it influences secondary stressors such as social life conflict and feeling of joy in caregiving. Separately, while there was no significant difference in loneliness between groups for the relationship to care recipient, the final Model 4 showed that caregivers who report more loneliness were not a child or other caregiver, thus suggesting spousal caregivers are more likely to report loneliness.

The primary stressors of the care recipient having dementia and the care recipients’ ADL needs indicated significant group differences on loneliness, but only ADL needs was significant in the final Model 4 predicting loneliness among caregivers. Based on the literature that dementia caregiving can be isolating (3), it was surprising that caregivers to people living with dementia had less loneliness compared to those caring for someone without dementia. Most people with 3+ ADL needs require extensive care and support, which can be overwhelming and time-intensive for caregivers. The analyses here revealed that caregivers to people with 3+ ADL needs reported more loneliness than caregivers to people with 0–2 ADL needs.

There were significant group differences for all of the caregiver-reported subjective measures representing secondary stressors. These variables were also significant when introduced in Model 3 of the hierarchical multivariate regression and in the final Model 4. The results reinforce that experiencing social conflicts with caregiving, not feeling joy from caregiving (5.69, *p* < 0.001), not feeling appreciation from the care recipient, and not feeling enough support are predictive of more loneliness among caregivers.

The mediator/caregiver support variables, which represent those caregiver services which may lessen experiences of loneliness, revealed interesting results. First, only about one-quarter of respondents reported attending caregiver training classes, receiving counseling, or attending support group services. While the NFCSP offers other services beyond these, further exploration is needed to understand the utilization and benefits of the NFCSP services. Second, only for the counseling service was there a significant difference in loneliness scores, with higher loneliness scores among those using counseling compares to those not using counseling, and with the use of counseling being a predictor of more loneliness among caregivers. While some may surmise that people accessing counseling would report lower levels of loneliness, there is also an argument to be made that the NFCSP counseling service is addressing those in need because of their experience with loneliness.

Secondary analyses of survey data include some limitations. First, the survey protocols for the 2019 NSOAAP – Family Caregiver Support module excludes some NFCSP caregivers from participating in the survey. The results presented here only demonstrate the experiences of caregivers to older adults. The experience of older relatives who are caring for children under the age of 18 or adults ages 18–59 with disabilities may not align with the results on loneliness and the role of caregiver supports. Second, these preliminary analyses only assessed direct effects on the outcome of loneliness and not the mediating effects of caregivers supports as outlined in the original SPMC (8). The direct effects found in this analysis reinforce the value of caregiver supports. Despite being limitations, the lack of information on older relative caregivers and testing for mediating effects of caregiver support are opportunities for future analysis.

By using the NSOAAP data, this study helps advance the goals of the National Strategy to Support Family Caregivers (6). By understanding the characteristics and experiences of caregivers reporting loneliness, this study contributes to the National Strategy’s “Goal 3: Strengthen services and supports for family caregivers” (p. 53) and “Goal 5: Expand data, research, and evidence-based practices to support family caregivers” (p. 79). Policies and programs focused on reducing caregiver loneliness should be accessible to all family caregivers but should prioritize outreach and engagement for groups more likely to experience loneliness, such as caregivers with poor self-reported health, spousal caregivers, caregivers to people with more ADL needs, and caregivers who experience negative caregiving strains (e.g., caregiving-related social conflicts). Although not explicit in the results, dementia caregivers often have the aforementioned characteristics and would benefit from services and programs that reduce loneliness. Masoud et al. (19) highlight the added value of virtual programming to support caregivers, and they note that the programming not only addresses loneliness but also education, resource sharing, and helping others – benefits for any type of caregiver. The findings overall underscore the importance of continuing and expanding efforts to address loneliness and related well-being outcomes among family caregivers.

## Data availability statement

Publicly available datasets were analyzed in this study. This data can be found at: https://agid.acl.gov/.

## Ethics statement

The studies involving humans were approved by Research Ethics & Integrity Program, Miami University. The studies were conducted in accordance with the local legislation and institutional requirements. Written informed consent for participation was not required from the participants or the participants’ legal guardians/next of kin in accordance with the national legislation and institutional requirements.

## Author contributions

HM: Conceptualization, Methodology, Writing – original draft, Writing – review & editing. CP: Conceptualization, Data curation, Formal analysis, Methodology, Writing – original draft, Writing – review & editing.
